# 

**Published:** 1911-02-15

**Authors:** 


CONTENTS

Event and Comment - - - - - - - - - - - - - - - - - - 55
Dental Anatomy, (Continued) - - - - *By N. S. Hoff, D.D.S.* 58
Electricity in Dental Practice, - *By L. E. Custer, A.M., D.D.S.* 66
Investments—What Not to Do, - - - - - *By Henry Hall.* 78
Educating the Educators, - - - *By E. A. Thomas, D.D.S.* 83
Indents - - - - - - - - - - - - - - - - - - - - - - - 89
Announcements - - - - - - - - - - - - - - - - - - - 103

